# Social media as a tool for oral health promotion: A systematic review

**DOI:** 10.1371/journal.pone.0296102

**Published:** 2023-12-19

**Authors:** Farzaneh Farrokhi, Zahra Ghorbani, Farid Farrokhi, Mahshid Namdari, Siavash Salavatian

**Affiliations:** 1 Department of Community Oral Health, School of Dentistry, Shahid Beheshti University of Medical Sciences, Tehran, Iran; 2 Department of Community Oral Health, School of Dentistry, Tehran University of Medical Sciences, Tehran, Iran; 3 Media Management Department, IRIB University, Tehran, Iran; King Faisal University, SAUDI ARABIA

## Abstract

Social media platforms are common means of sharing information, personal experiences, and lifestyle. They can also be utilized as cost-effective methods for individuals to acquire health information and promote oral health. The purpose of the present study was to systematically review the current literature on the interventions taken through social media for promoting lay people’s oral health. This systematic review (PROSPERO: CRD42023395005) followed the preferred reporting items for systematic reviews and meta-analyses (PRISMA) 2020 guidelines. A comprehensive search was conducted in four electronic databases (PubMed, Scopus, Embase, and Cochrane Library) for relevant articles published between 2012 and 2023. Data such as study design, sample size, follow-up duration, utilized social media platforms and main findings were extracted from the eligible studies. The quality of the studies included in the systematic review was evaluated by the quality assessment tools for intervention studies recommended by the National Collaborating Centre for Methods and Tools. Out of the 1934 records identified in the initial search, 10 studies met the inclusion criteria and were included in the qualitative synthesis. These studies comprised seven randomized control trials, one field trial and two quasi-experimental. Various social media platforms, including Telegram, Instagram, YouTube, WhatsApp and Snapchat, were used for communication with patients. Some studies solely utilized social media interventions, while others combined online and traditional interventions. The quality assessment categorized 30% of the studies as “strong”, 50% as “moderate”, and the remaining as “weak”. The implementation of social media interventions positively influenced multiple aspects of oral health among the laypeople. Online platforms such as YouTube, WhatsApp, Instagram, and Telegram can be effectively utilized to promote oral health among patients.

## Introduction

Social media can be described as "a group of internet-based applications that are built on Web 2.0.". One of the pivotal concepts underlying Web 2.0 is "individual production and user-generated content" [1, 2]. Social media has gained significant popularity in recent decades, allowing users to create, share, and participate in social networks [3]. As of February 2023, there were approximately 4.95 billion social media users worldwide, accounting for 59.4% of the global population [4]. The most commonly used social networking platforms include Facebook, YouTube, WhatsApp, Instagram, and WeChat [5].

Multitudinous online activities have been designed for medical support and increasing the health knowledge. Social media, in particular, offers a convenient and accessible platform for individuals seeking healthcare information [6]. Oral diseases affect 3.5 billion of the worldwide population and cases a high burden of diseases [7]. Dental health professionals can leverage social media as an opportunity to provide public health education and information about oral health issues to a large number of patients [8]. Social media is able to increase the degree of knowledge in people of all ages, and it can be used as a promising clinical tool for the prevention and promotion of oral health [9, 10].

While there have been several studies investigating the impact of social media on dentistry, there is limited information available on the effectiveness of online interventions through social media aimed at preventing diseases and promoting oral health among laypeople. Clinicians can benefit from familiarizing themselves with the interventions described in the literature and evaluating the reported outcomes. This will enable them to make informed decisions using the cost-effective and accessible tool of social media. Therefore, the objective of this study is to systematically review the current evidence regarding social media-based interventions designed for disease prevention and oral health promotion among laypeople.

## Materials and methods

This systematic review was registered at PROSPERO (international prospective register of systematic reviews) (CRD42023395005), and was carried out in accordance with the PRISMA (preferred reporting items for systematic reviews and meta-analyses) 2020 statement.

### Eligibility criteria

This systematic review aims to address the following PIO question: “What is the effect of social media on prevention and promotion of oral health compared with conventional strategies among lay people of any age without disability or systematic diseases?”. The review encompasses (P) laypeople from diverse backgrounds, including different age groups, genders, socioeconomic statuses, ethnicities without any disability or systematic diseases. The focus is on (I) social media-based interventions that employ a group approach for information dissemination. The desired outcomes include (O) any changes in oral health status, knowledge, beliefs, and attitudes resulting from social media interventions. This includes short and long-term effects, regardless of the duration of follow-up. The full-text articles from 2012 to 2023 focus on randomized controlled trials (RCTs), quasi-randomized trials and field trials were included in the current study. Studies that did not specifically target oral disease prevention or oral health promotion, were not based on social media approaches, had an intervention on oral health promotion of people with systematic disease or disability or were published in languages other than English were excluded. The conference proceedings, editorials, commentaries, review articles, pilot studies and all the studies that assessed the quality of the content of social media were excluded.

### Search strategy

In consultation with a community oral health specialist and a media management specialist, following search queries were designed: (“social media” OR “social network*” OR “Social medium*” OR web2.0* OR web3.0 OR co-creation OR Twitter OR tweet* OR snapchat* OR Instagram* OR Facebook OR YouTube OR TikTok OR Clubhouse OR LinkedIn OR blog* OR weblog* OR microblog* OR tumbler OR vimeo) AND (“oral health” OR “dental health services” OR “dental caries” OR periodont* OR gingiv* OR dent* OR tooth OR “oral hygienist” OR “oral cancer”). The search was performed from 1st January 2012 until 25^th^ Augst 2023, seeking papers published in English language. Four electronic databases, including PubMed, Scopus, Embase, and Cochrane Library, were searched thoroughly. The search was limited to the title and abstract in PubMed and Embase, while the title, abstract, and keyword fields were searched in Cochrane Library and Scopus. Additionally, a manual search was performed to identify any potentially missed studies by examining the reference lists of included studies and previously published reviews.

### Screening process

The results of the databases were exported into EndNote (EndNote 2015. Version X7. Thomson). Duplications were removed using EndNote-based methods as reported previously [11], and double-checked manually. After removing the duplicated records, two trained researchers (Fz.F and F.F) conducted the screening process independently. Following their training, reviewers independently double screened the title and abstracts of the same records until they reached 85% inter‐rater reliability rate for include/exclude decisions. In the first stage the titles and abstracts of the remaining studies were screened against the inclusion and exclusion criteria. Those that were relevant and met the inclusion criteria were flagged for a full‐text review in the second stage of the selection process. If it was not clear from the abstract whether the paper met the inclusion criteria, it was included for full-text screening. Subsequently, full texts of the potentially relevant articles were thoroughly evaluated by two researchers (Fz.F and F.F). In case of any disagreement, the final decision was made after consulting a third person (Z.G).

For each selected article, data such as study design, sample size, follow-up duration, utilized social media platforms and main findings were extracted.

### Quality assessment

The quality of the studies included in the systematic review was evaluated using the “quality assessment tools for quantitative studies”, recommended by the National Collaborating Centre for Methods and Tools [12]. This tool is accompanied by a detailed dictionary that provides explicit guidance for decision-making within each domain (S1 File). The evaluation covered domains including selection bias, study design, confounding factors, blinding, data collection methods, the integrity of the intervention, method of analysis, withdrawal and dropout. The overall quality of each study was categorized as “weak”, “moderate”, and “strong”. Studies devoid of any “weak” rating and receiving at least four “strong” ratings were considered “strong”. Studies with one “weak” rating and three or less “strong” ratings were qualified as “moderate”, while studies with two or more “weak” ratings were regarded as “weak” ones. Fz.F and F.F read and rated the full texts, and in case of disagreement, M.N made the final decision.

## Results

### Study selection

**Fig 1:**
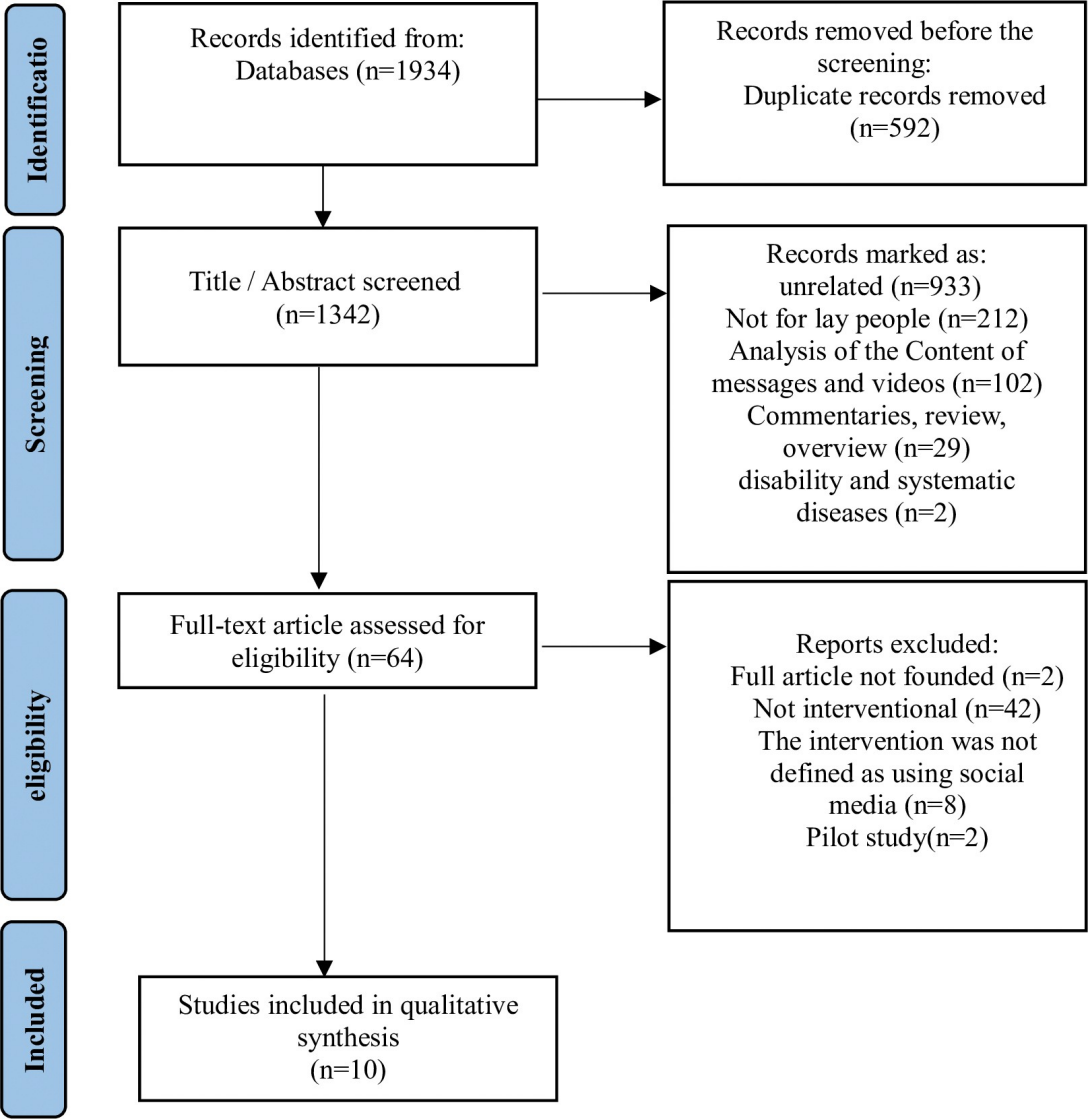
Flow diagram of the screening process according to the PRISMA [13].

After conducting the electronic search, a total of 1,934 records were identified. Following the removal of duplicates, 1342 papers remained. Through title and abstract screening, 64 studies were identified as potentially relevant to education, awareness about treatment, prevention, and promotion of oral health in laypeople. Upon full-text assessment, 54 studies were excluded, and 10 articles that met the inclusion/exclusion criteria were included in the qualitative synthesis (Fig 1).

### Study characteristics

An overview of the included studies, containing their characteristics, study designs, follow-up durations, and other relevant information, can be found in Table 1. Regarding the study design, seven RCTs [14–20], a field trial [21] and two quasi-experimental studies [22, 23] were included in this systematic review. Eight studies employed a design in which two or more groups were compared [14–21], and the rest reported changes before and after the intervention within a single study group [22, 23]. Among the reviewed papers, five articles utilized Telegram as a platform for delivering oral health information through channels and groups [16, 19, 21–23]. Two studies employed Instagram [14, 15], and one used YouTube [18]. Two study used a WhatsApp group [17, 20] and one study used Snapchat [20]. The sample sizes ranged from 40 to 791 participants per study, and the age range of the participants varied from newborns to 71 years old. The study durations ranged from 1 to 24 months.

**Table 1:** Summary of included studies in systematic review.

	Author (Year, country)	Study type	Aim	sample size/ Participants	Follow up	Social media	Outcomes	Main findings
1	Zareban et al. (Iran, 2022)	Randomized controlled trial	Investigating the effect of the oral health education program presented in Telegram on self-efficacy, perceived benefits and barriers, gingival index, motivational beliefs and tooth cleaning behavior in students with gingivitis.	160 students Control:(n = 80) Intervention:(n = 80) Inclusion: age ranged from 12 to 19	24 weeks	Telegram	• Dental cleaning behavior • Perceived self-efficacy • Perceived benefits • Perceived barriers • Motivational beliefs • Gingival index	Significant differences between the intervention and the control groups in the mean scores of perceived self-efficacies (p = 0.01), Perceived benefits (p = 0.01) motivational beliefs (p = 0.01), Gingival index (p = 0.01) after the intervention.
2	Deghatipour et al. (Iran, 2022)	Field trial	Evaluating the effectiveness of some oral health interventions on pregnant women dental caries.	439 mothers from pregnancy up to 2 years after delivery Intervention:(n = 239) A: Comprehensive method including all following methods together (n = 74) B: Group discussion by dentists (n = 59) C: Face to face education by primary health care providers (n = 53) D: Social network applications (n = 53) Control:(n = 215) Routine maternal and oral health care (n = 215) inclusion: pregnant women (15 years and older) in the second/third trimester of pregnancy	24 months	Telegram	• Dental care behavior • DMFT	The mean D significantly decreased nearly 1unit at same period (P<0.05). Most and least dental caries changes were in comprehensive intervention group and social network intervention group compared to other intervention groups.
3	Tahani et al. (Iran, 2022)	Interventional quasi-experimental	Investigate the effect of an Oral Health Promoting School (OHPS) model on children’s oral health	354 primary school students and their parents Inclusion: second-grade 7–8 years old students and their parents	5 months	Telegram	• knowledge • Attitude	The mean pre-test knowledge (7.8±1.7) was increased significantly in three schools after program, p<0.001. In the post-test, girls gained significantly higher scores (9.61±1.98 vs. 9.06±1.4, p = 0.025). Knowledge score of the parents attending both sessions was higher. Practice of the parents regarding the use of fluoridated tooth-paste was significantly improved (p<0.001).
4	Bates et al. (USA, 2012)	Interventional quasi-experimental	Investigating an online approach to promote awareness of oral health messages targeting pregnant women and whether this type of health messaging affects oral health knowledge and beliefs.	55 individuals Inclusion: general public (including pregnant women)	2 months	You tube	• Knowledge • Belief	Increase in knowledge from pre- to post commercial viewing
5	Sivrikaya et al. (Turky, 2021)	Randomized, double-blinded and controlled trial	Assessing the effects of dentist–patient communication via social media on dental anxiety and to determine the appropriate timing of such communications.	Patients who underwent impacted lower right third molar extraction Intervention: A-only after (n = 36) B-before and after the operation (n = 36) C-only before (n = 35) Control: received no communication on social media (n = 36) Inclusion: f patients who underwent impacted lower right third molar extraction	-	Instagram	• Dental anxiety	The results showed that the post-op values of control group had higher anxiety scores than the before and after the operation and only before according to VAS (p < 0.05). Within the groups, the anxiety levels showed a decreasing trend after surgery according to MDAS and VAS scores (p < 0.05). The results of this study suggest that communication with patients before the operation is sufficient to reduce their dental anxiety.
6	Scribante et al. (Italy, 2021)	Single-center, parallel, randomized controlled trial	Investigating the effectiveness of Instagram in improving oral hygiene compliance and knowledge in young orthodontic patients compared to traditional chairside verbal instructions.	40 patients having fixed appliances Intervention: (n = 20) Verbal instructions+ multimedia contents Control: (n = 20) Verbal instructions Inclusion: presence of fixed orthodontic appliances on both arches during the following 6 months, age between 13 and 19 years old, no mental disabilities	1,3 and 6 months	Instagram	• Bleeding index (BI) • Modified gingival index (MGI) • Plaque index (PI) • knowledge	In both groups, BI, MGI, and PI significantly decreased (p < 0.05) at T1 (means control group: BI 0.26 ± 0.22, MGI 0.77 ± 0.36, PI 0.53 ± 0.20; means test group: BI 0.24 ± 0.22, MGI 0.65 ± 0.46, PI 0.49 ± 0.21) compared to baseline (means control group: BI 0.56 ± 0.27,MGI 1.23 ± 0.41, PI 0.87 ± 0.23; means test group: BI 0.54 ± 0.26, MGI 1.18 ± 0.39, PI 0.93 ± 0.20) but no significant differences in clinical measures were showed between T1, T2, and T3 (p > 0.05) (intragroup differences).demonstrated significant improvements in knowledge with respect to controls comparing scores at T0 and T3 (p < 0.05)
7	Scheerman et al. (Iran, 2020)	Three-arm randomized-controlled trial	Investigating the efficacy of a theory-based program using an online social media platform (Telegram) to promote good oral hygiene behavior among Iranian adolescents.	Interventional group: adolescent only intervention group (A, n = 253) adolescent and mother intervention group (A + M; n = 260) control group (n = 278) Inclusion: adolescents age 17–19, not engaged in other oral health education or research program; willing to participate and provided written informed consent before entry to the study; no physical and/or cognitive disabilities impeding the ability to perform	1 and 6 months	Telegram	• Psychosocial variables • Toothbrushing behavior • Visual Plaque Index • Community Periodontal Index	Increases in adolescent toothbrushing at the one- and six-month follow-ups in both intervention groups compared to the control group were observed. Adolescents in the A + M group showed significant greater improvements in their toothbrushing behavior, Visual Plaque Index, and Community Periodontal Index scores than adolescents in the A group. Improvements to toothbrushing social cognitions were also observed.
8	Zotti et al. (Italy, 2016)	Randomized controlled trial	Evaluating the influence of an app-based approach in a protocol for domestic oral hygiene maintenance in a group of adolescent patients wearing fixed multibracket appliances.	Eighty adolescent patients scheduled to start an orthodontic multibracket treatment Interventional:(n = 40) Control:(n = 40) Inclusion: adolescent patients who were scheduled to start orthodontic multibracket treatment	3,6,9 and 12 months	WhatsApp	• Plaque index (PI) • Gingival index (GI) • White spots (WS) • Caries presence	After 3-month, interventional group had significantly lower values of both PI and GI and a lower incidence of new WS and caries, compared with the control group.
9	Al-Silwadi et al. (United Kingdom,2015)	Single center parallel-group randomized controlled trial	Assessing whether provision of audiovisual information on the YouTube (Google, San Bruno, Calif) Web site to orthodontic patients undergoing fixed appliance treatment results in improved patient knowledge when compared with conventional methods of information provision.	intervention (n = 33) view a 6-minute YouTube video + verbal and written information control (n = 34) verbal and written information Inclusion: Orthodontic patients were 13 years of age and over, with no history of orthodontic treatment	6 to 8 weeks	YouTube	• Knowledge	Those who completed the trial in the intervention group demonstrated significantly greater improvements in knowledge than did those in the control group. Ethnicity had a statistically significant effect on improvement in knowledge
10	Aboalshamat et al. (Saudi Arabia, 2023)	Single-blinded parallel group randomized controlled trial	Evaluating the effects of social media (Snapchat) dissemination of health-promoting interventions on knowledge of oral health during pregnancy among pregnant women	68 pregnant women Snap chat group (n = 34) Written flyer group (n = 34) Inclusion: pregnant women	1month	Snapchat, WhatsApp	• Knowledge	Total knowledge scores in the social media group significantly increased immediately following the intervention

### Quality assessment results

The overall quality of three studies was categorized as “strong” [14, 16, 17, 21], five studies as “moderate” [18–22], and two studies as “weak” [15, 23] (Table 2). Regarding the representativeness of the target population, five studies were rated as “strong” in terms of participant selection quality, while the remaining studies were assessed as “moderate”. Eight studies, including randomized controlled trials (RCTs), field trials, were deemed to have a “strong” study design, while two studies were considered “moderate”. The majority of studies demonstrated control over confounding factors and were therefore classified as “strong”. Due to the nature of the interventions, blinding was not feasible in most studies, although many reported blinding of outcome assessors. Only one study considered blindness for both assessors and participants. The quality of data collection methods was determined as “strong” for four studies, “moderate” for two studies and “weak” for the remaining studies. Seven studies provided detailed information on participant withdrawal and dropout, earning a rating of “strong”, while two studies were classified as “weak”, and one study received a rating of “moderate”.

**Table 2:** Quality assessment results.

	Selection	Study Design	Confounders	Blinding	Data Collection Method	Dropout and Withdrawal	Global Rating
**Zareban et al. (Iran,2022)**	Strong	Strong	Strong	Moderate	Strong	Weak	Moderate
**Deghatipour et al. (Iran,2022)**	Moderate	Strong	Strong	Moderate	Moderate	Strong	Moderate
**Tahani et al. (Iran,2022)**	Moderate	Moderate	Strong	Weak	Strong	Moderate	Moderate
**Bates et al. (USA,2012)**	Moderate	Moderate	Strong	Weak	Weak	Weak	Weak
**Sivrikaya et al. (Turky,2021)**	Moderate	Strong	Strong	Strong	Strong	Strong	Strong
**Scribante et al. (Italy,2021)**	Moderate	Strong	Weak	Moderate	Weak	Strong	Weak
**Scheerman et al. (Iran,2020)**	Strong	Strong	Strong	Moderate	Moderate	Strong	Strong
**Zotti et al. (Italy,2016)**	Strong	Strong	Strong	Moderate	Strong	Strong	Strong
**Al-Silwadi et al. (United Kingdom, 2015)**	Strong	Strong	Strong	Moderate	Weak	Strong	Moderate
**Aboalshamat et al. (SaudiArabia,2023)**	Moderate	Strong	Strong	Moderate	Weak	Strong	Moderate

Regarding intervention integrity, all the studies provided details on the theoretical basis, intervention frequency, compliance, and methods of follow-up contacts for participants. Two studies did not assess the consistency of interventions [19, 22], and the rest did not provide a clear explanation on this matter. Two studies mentioned the possibility of contamination [18, 21]. Eight studies utilized bivariate analysis [14, 15, 17, 19–23], while three studies employed multivariate analysis [16, 18, 21]. Most of the studies used appropriate statistical methods for data analysis [14, 16–22]. Out of all the included studies, three studies did not have any lost to follow-up [15, 17, 19], while in the others that had instances of lost to follow-up [14, 16, 18, 20–23], only one study explicitly stated that the analysis was performed based on intention to treat [16], while the others did not clarify.

### Interventions

The interventions reported in the included studies can be categorized into two groups: (1) interventions that combined social media with traditional education methods, and (2) interventions that solely relied on social media to enhance awareness and improve oral health.

### Social media alongside traditional interventions

Two studies focused on orthodontic patients and evaluated the impact of using social media in addition to standardized oral hygiene instructions. Both the intervention and control groups received verbal or written instructions, while the intervention group also received education through social media. Both studies demonstrated a significant increase in oral health knowledge among the intervention group compared to the control group [15, 18]. However, there were no significant differences between the groups in terms of bleeding index, modified gingival index, and plaque index in one of the studies [15]. Another study assessed the role of social media in maintaining oral health in adolescents with fixed orthodontic appliances. After six months, the intervention group exhibited significantly lower plaque index, better gingival index, and a lower incidence of new white spot lesions/caries compared to the control group [17].

One study used a Telegram channel to deliver oral health education programs, including audio, video, and text messages related to nutrition and behavior. This study concluded that the use of social media alone had an insignificant effect on preventing dental caries among pregnant women when compared to other interventions such as face-to-face education or group discussions, or a combination of interventions [21].

Another study incorporated social media, along with educational sessions, pamphlets, tooth-brushing diaries, and at-home assignments, to deliver educational texts and videos. The study reported that the education positively influenced students’ awareness, knowledge, and attitudes. However, the impact of social media as an individual factor was not separately assessed in this study [22].

### Social media interventions

Two studies focused on developing educational programs using social media to improve oral hygiene behavior among adolescents, employing behavior change models [19, 16]. These studies included two intervention groups: adolescents alone and adolescents with their mothers. The findings indicated that the inclusion of mothers in social media groups significantly enhanced brushing behavior, visual plaque index, and periodontal index compared to adolescents alone [16]. Both studies reported statistically significant differences in clinical, behavioral, and psychological indices between the intervention and control groups [16, 19].

Another study investigated the effects of dentist-patient communication via social media on dental anxiety. In the intervention group, social media was utilized to address patients’ questions and alleviate dental anxiety in patients undergoing impacted tooth extraction under local anesthesia. The study revealed that anxiety scores in the control group were significantly higher compared to the intervention group [14].

two study examined the impact of social media on oral health knowledge and beliefs among pregnant women. Both of the studies demonstrated that social media effectively expanded the oral health knowledge of pregnant women [20, 23].

### Types of outcomes measured

Six studies assessed the objective oral health using gingival index [17, 19], modified gingival index [15], decayed, missing, and filled teeth (DMFT) [21], bleeding index [15], plaque index [15–17], number of decayed teeth [17, 18, 21], community periodontal index [16], and white spots [17]. Behavioral changes, including dental cleaning behavior [16, 19, 21, 22], perceived self-efficacy, perceived benefits/barriers, motivational beliefs [19], and psychosocial variables [16], were assessed in different studies. Dental anxiety [14], knowledge [14, 20, 22] and attitude about oral health [22] were other measured outcomes.

## Discussion

In recent years, the widespread use of social networks has provided an opportunity for enhanced communication, either independently or in conjunction with traditional methods, to improve oral health. Most studies solely utilizing social media were rated as “moderate” or “strong” in quality, while the majority of studies combining social media with traditional methods demonstrated “moderate” quality. Few studies received a “strong” rating for blinding and data collection domains. It is important to note that blinding was not feasible in many studies due to the nature of the interventions, but data collection methods could be strengthened by utilizing valid and reliable tools.

Performing meta-analysis was not feasible because of heterogeneity in study designs, sample population, outcome measures, and interventions [24]. Due to insufficient data and variations in the results, no definitive conclusions could be drawn regarding the preferred method. However, based on the available evidence, it can be concluded that utilizing social media is a positive step towards improving oral health.

Selecting the appropriate platform is a crucial factor in the success of health promotion interventions. Audiovisual social media platforms such YouTube may be more effective in promoting oral health compared to text-based formats [18]. Visual content is processed more rapidly by the brain compared to text, and research indicates that the majority of information processed by the brain is visual [25]. Currently, Facebook and YouTube are the most popular social networks globally, each with nearly three billion users [5]. Additionally, a literature review found that Facebook was the most commonly used social media site for obtaining health information on various topics, including vaccinations, alcohol or drug abuse, physical activity, and breastfeeding [26]. However, when designing oral health promotion interventions, it is important to consider the popularity of social media platforms among the target community.

Teenagers today spend a significant amount of time using their smartphones [27]. Thus, implementing behavior change theories through popular social media platforms can have a positive impact on oral health behaviors among adolescents [15, 18]. Social media can be a valuable tool in increasing knowledge about dental care and orthodontic appliances among orthodontic patients, who predominantly consist of teenagers requiring information about appliances and adapted brushing and flossing techniques [15, 17, 18]. Utilizing social media for patient education saves time for clinicians and allows patients to access instructions whenever needed, enhancing their comfort and independence. This aligns with another study that highlights the positive influence of interventions conducted through social networking websites on health behavior outcomes [28]. Furthermore, involving mothers alongside their adolescents in interventions can bring additional benefits to teenagers’ oral health, as parents, especially mothers, play a crucial role in supporting and encouraging their children towards a healthy lifestyle and good oral health behaviors [29].

Implementing educational interventions during pregnancy and after childbirth through social media or a combination of social media and traditional methods (e.g., face-to-face education and group discussions led by dentists) may yield the best outcomes in promoting the oral health of both mothers and their children [20, 21, 23]. This finding is consistent with another study in this systematic review, which emphasizes the role of mothers in their children’s oral health [16]. Providing educational interventions during and after pregnancy contributes to a safe pregnancy and improved maternal well-being [30, 31]. However, it should be noted that some mothers may not frequently use social media due to concerns about the potential harms of internet and mobile phone usage on the fetus. Therefore, utilizing a combination of methods ensures that a wide range of pregnant women receive prenatal education [32].

### Limitations

Considering the different inclusion criteria in these studies, the results cannot be generalized to all groups.

### Conclusions

In conclusion, although social media offers efficiency and convenience for interventions aimed at oral health prevention and promotion, its utilization is not yet widespread. While social media can enhance knowledge transfer and improve content accessibility, the success of interventions depends on evidence-based approaches and individual participation. Online platforms such as Facebook, YouTube, WhatsApp, Instagram, and Telegram can serve as appropriate tools for these interventions due to their popularity among global users. However, it is important to note that there is no single method or platform that can be deemed universally suitable for all regions. Nevertheless, audio-visual platforms may be more effective than text-based social media in promoting oral health.

Given the integration of social media and new technologies into people’s lives, the significance of these tools in shaping oral health behaviors is undeniable. The rise of user-generated content and participatory engagement strategies will enable a more inclusive and collaborative approach to oral health promotion. Collaborations between oral health professionals, organizations, and influencers on social media will amplify credibility and reach. However, these advancements come with ethical considerations, demanding vigilance in adhering to privacy regulations and combatting misinformation.

Considering these changes, it is crucial for researchers and practitioners to recognize and address research gaps. Future studies should explore unexplored areas to gain a deeper insight of the intersection between social media and oral health promotion.

## Supporting information

S1 Checklist PRISMA 2020 checklist. (DOCX)

S1 Table Summary of included articles in systematic review. (DOCX)

S1 File Quality assessment tool and quality assessment dictionary. (PDF)

S2 File Quallity assessment of the studies. (PDF)

S3 File Total studies. (XLSX)

S4 File Duplicate removal. (XLSX)

S5 File Final selected studies. (XLSX)
