# Supplementary material for: Social media as a tool for oral health promotion: A systematic review
Source: PLoS One. 2023 Dec 19;18(12):e0296102. doi: 10.1371/journal.pone.0296102 (PMC10729958; doi:10.1371/journal.pone.0296102)
Supplement: S1 Table — (DOCX) [file pone.0296102.s002.docx]

**table 1**. **Summary of included studies in systematic review**

|  | **Author**  **(Year, country)** | **Study type** | **Aim** | **sample size/ Participants** | **Follow up** | **Social media** | **Outcomes** | **Main findings** |
| --- | --- | --- | --- | --- | --- | --- | --- | --- |
| 1 | Zareban et al. (Iran,2022) | Randomized controlled trial | Investigating the effect of the oral health education program presented in Telegram on self-efficacy, perceived benefits and barriers, gingival index, motivational beliefs and tooth cleaning behavior in students with gingivitis. | 160 students  Control:(n=80)  Intervention:(n=80)  Inclusion: age ranged from 12 to 19 | 24 weeks | Telegram | - Dental cleaning behavior  - Perceived self-efficacy  - Perceived benefits  - Perceived barriers  - Motivational beliefs  - Gingival index | Significant differences between the intervention and the control groups in the mean scores of perceived self-efficacies (p=0.01), Perceived benefits (p=0.01) motivational beliefs (p=0.01), Gingival index (p=0.01) after the intervention. |
| 2 | Deghatipour et al. (Iran,2022) | Field trial | Evaluating the effectiveness of some oral health interventions on pregnant women dental caries. | 439 mothers from pregnancy up to 2 years after delivery  Intervention:(n=239 )A: Comprehensive method including all following methods together (n=74)  B: Group discussion by dentists (n=59)  C: Face to face education by primary health care providers (n=53)  D: Social network applications (n=53)  Control:(n=215) Routine maternal and oral health care (n=215) inclusion: pregnant women (15 years and older) in the second/third trimester of pregnancy | 24 months | Telegram | - Dental care behavior  - DMFT | The mean D significantly decreased nearly 1unit at same period (P<0.05). Most and least dental caries changes were in comprehensive intervention group and social network intervention group compared to other intervention groups. |
| 3 | Tahani et al. (Iran,2022) | Interventional quasi-experimental | Investigate the effect of an Oral Health Promoting School (OHPS) model on children’s oral health | 354 primary school students and their parents  Inclusion: second-grade 7–8 years old students and their parents | 5 months | Telegram | - knowledge  - Attitude | The mean pre-test knowledge (7.8±1.7) was increased significantly in three schools after program,  p<0.001.  In the post-test, girls gained significantly higher scores (9.61±1.98 vs. 9.06±1.4, p=0.025).  Knowledge score of  the parents attending both sessions was higher.  Practice of the parents regarding the use of fluoridated tooth-paste  was significantly improved (p<0.001). |
| 4 | Bates et al. (USA,2012) | Interventional quasi-experimental | Investigating an online approach to promote awareness of oral health messages targeting pregnant women and whether this type of health messaging affects oral health knowledge and beliefs. | 55 individuals  Inclusion: general public (including pregnant women) | 2 months | You tube | - Knowledge  - Belief | Increase in knowledge from pre- to post commercial viewing |
| 5 | Sivrikaya et al. (Turky,2021) | Randomized, double-blinded and controlled trial | Assessing the effects of dentist–patient communication via social media on dental anxiety and to determine the  appropriate timing of such communications. | Patients who underwent impacted lower right third molar extraction  Intervention:  A-only after (n = 36)  B-before and after the operation (n = 36)  C-only before (n = 35)  Control: received no communication on social media (n = 36)  Inclusion: f patients who underwent impacted lower right third molar extraction | - | Instagram | - Dental anxiety | The results showed that the post-op values of control group had higher anxiety scores than the before and after the operation and only before according to VAS (p < 0.05).  Within the groups, the anxiety levels showed a decreasing trend after surgery according to MDAS and VAS scores (p < 0.05).  The results of this study suggest that communication with patients before the operation is sufficient to reduce their dental anxiety. |
| 6 | Scribante et al. (Italy,2021) | Single-center, parallel, randomized controlled trial | Investigating the effectiveness of Instagram in improving oral hygiene compliance and knowledge in young orthodontic patients compared to traditional chairside verbal instructions. | 40 patients having fixed appliances  Intervention: (n = 20)  Verbal instructions+ multimedia contents  Control: (n = 20)  Verbal instructions  Inclusion: presence of fixed orthodontic appliances on both arches during the following 6 months, age between 13 and 19 years old, no mental disabilities | 1,3 and 6 months | Instagram | - Bleeding index (BI)  - Modified gingival index (MGI)  - Plaque index (PI)  - knowledge | In both groups, BI, MGI, and PI significantly decreased (p < 0.05) at T1 (means control group: BI 0.26 ± 0.22, MGI 0.77 ± 0.36, PI 0.53 ± 0.20; means test group: BI 0.24 ± 0.22, MGI 0.65 ± 0.46, PI 0.49 ± 0.21) compared to baseline (means control group: BI 0.56 ± 0.27,MGI 1.23 ± 0.41, PI 0.87 ± 0.23; means test group: BI 0.54 ± 0.26, MGI 1.18 ± 0.39, PI 0.93 ± 0.20) but no significant differences in clinical measures were showed between T1, T2, and T3 (p > 0.05) (intragroup differences).demonstrated significant improvements in knowledge with respect to controls comparing scores at T0 and T3 (p < 0.05) |
| 7 | Scheerman et al. (Iran,2019) | Three-arm randomized-controlled trial | Investigating the efficacy of a theory-based program using an online social media platform (Telegram) to promote good oral hygiene behavior among Iranian adolescents. | Interventional group:  adolescent only intervention group (A, n = 253)  adolescent and mother intervention group (A + M; n = 260)  control group (n = 278)  Inclusion: adolescents age 17-19, not engaged in other oral health education or research program; willing to participate and provided written informed consent before entry to the study; no physical and/or cognitive disabilities impeding the ability to perform | 1 and 6 months | Telegram | - Psychosocial variables  - Toothbrushing behavior  - Visual Plaque Index  - Community Periodontal Index | Increases in adolescent toothbrushing at the one- and six-month follow-ups in both intervention groups compared to the control group were observed. Adolescents in the A + M group showed significant greater improvements in their toothbrushing behavior, Visual Plaque Index, and Community Periodontal Index scores than adolescents in the A group.  Improvements to toothbrushing social cognitions were also observed. |
| 8 | Zotti et al. (Italy,2016) | Randomized controlled trial | Evaluating the influence of an app-based approach in a protocol for domestic oral hygiene maintenance in a group of adolescent patients wearing fixed multibracket appliances. | Eighty adolescent patients scheduled to start an orthodontic multibracket treatment  Interventional:(n=40)  Control:(n=40)  Inclusion: adolescent patients who were scheduled to start orthodontic multibracket treatment | 3,6,9 and 12 months | What’s App | -Plaque index (PI)  -Gingival index (GI)  -White spots (WS)  -Caries presence | After 3-month, interventional group had significantly lower values of both PI and GI and a lower incidence of new WS and caries, compared with the control group. |
| 9 | Al-Silwadi et al. (United Kingdom,2015) | Single center parallel-group randomized controlled trial | Assessing whether provision of audiovisual information on the YouTube (Google, San Bruno, Calif) Web site to orthodontic patients undergoing fixed appliance treatment results in improved patient knowledge when compared with conventional methods of information provision. | intervention (n = 33)  view a 6-minute YouTube video + verbal and written information  control (n = 34)  verbal and written information  Inclusion: Orthodontic patients were 13 years of age and over, with no history of orthodontic treatment | 6 to 8 weeks | YouTube | -Knowledge | Those who completed the trial in the intervention group demonstrated significantly greater improvements in knowledge than did those in the control group.  Ethnicity had a statistically significant effect on improvement in knowledge |
| 10 | Aboalshamat et al. (Saudi Arabia,2023) | Single-blinded parallel group randomized controlled trial | Evaluating the effects of social media (Snapchat) dissemination of health-promoting interventions on knowledge of oral health during pregnancy among pregnant women | 68 pregnant women  Snap chat group (n=34)  Written flyer group (n=34)  Inclusion: pregnant women | 1month | Snapchat, WhatsApp | -Knowledge | Total knowledge scores in the social media group significantly increased immediately following the intervention |
